# MicroRNA miR-29c Down-Regulation Leading to De-Repression of Its Target DNA Methyltransferase 3a Promotes Ischemic Brain Damage

**DOI:** 10.1371/journal.pone.0058039

**Published:** 2013-03-13

**Authors:** Gopal Pandi, Venkata P. Nakka, Ashutosh Dharap, Avtar Roopra, Raghu Vemuganti

**Affiliations:** 1 Department of Neurological Surgery, University of Wisconsin, Madison, Wisconsin, United States of America; 2 Department of Neuroscience, University of Wisconsin, Madison, Wisconsin, United States of America; University of Queensland, Australia

## Abstract

Recent studies showed that stroke extensively alters cerebral microRNA (miRNA) expression profiles and several miRNAs play a role in mediating ischemic pathophysiology. We currently evaluated the significance of miR-29c, a highly expressed miRNA in rodent brain that was significantly down-regulated after focal ischemia in adult rats as well as after oxygen-glucose deprivation in PC12 cells. Bioinformatics indicated that DNA methyltransferase 3a (DNMT3a) is a major target of miR-29c and co-transfection with premiR-29c prevented DNMT3a 3′UTR vector expression. In PC12 cells, treatment with premiR-29c prevented OGD-induced cell death (by 58±6%; p<0.05). Furthermore, treatment with antagomiR-29c resulted in a 46±5% cell death in PC12 cells. When rats were treated with premiR-29c and subjected to transient focal ischemia, post-ischemic miR-29c levels were restored and the infarct volume decreased significantly (by 34±6%; p<0.05) compared to control premiR treated group. DNMT3a *si*RNA treatment also significantly curtailed the post-OGD cell death in PC12 cells (by 54±6%; p<0.05) and decreased the post-ischemic infarct volume in rats (by 30±5%; p<0.05) compared to respective control *si*RNA treated groups. The miR-29c gene promoter showed specific binding sites for the transcription factor REST and the miR-29c promoter vector expression was curtailed when cotransfected with a REST expressing plasmid. Furthermore, treatment with REST *si*RNA prevented the post-ischemic miR-29c down-regulation and DNMT3a induction in PC12 cells and curtailed ischemic cell death (by 64±9%; p<0.05) compared to control *si*RNA treatment. These studies suggest that miR-29c is a pro-survival miRNA and its down-regulation is a promoter of ischemic brain damage by acting through its target DNMT3a. Furthermore, REST is an upstream transcriptional controller of miR-29c and curtailing REST induction prevents miR-29c down-regulation and ischemic neuronal death.

## Introduction

MicroRNAs (miRNAs) are 17 to 23 nucleotides long, evolutionarily conserved non-coding RNAs that control protein translation by binding to the complementary seed sequences in the 3′UTRs of mRNAs [1]. We and others reported that ischemia significantly alters cerebral miRNAome and specific miRNAs modulate either neuroprotection or neuronal death in the post-ischemic brain [2], [3], [4], [5], [6], [7], [8], [9], [10], [11]. Our studies showed that miR-29c is one of the highly expressed miRNAs in rat brain that was down-regulated in a sustained manner during the acute phase (2 h to 3 days) after focal ischemia [3]. We currently evaluated the functional significance of miR-29c in ischemic cell death under *in vitro* (oxygen-glucose deprivation; OGD in PC12 cells) and *in vivo* (transient middle cerebral artery occlusion; MCAO in adult rats) conditions. As *in silico* analysis showed DNA methyltransfease 3a (DNMT3a) as a robust target of miR-29c, we tested the role of DNMT3a in mediating the post-ischemic effects of miR-29c. We further evaluated if repressor element 1 silencing transcription factor (REST) *aka* neuron-restrictive silencing factor (NRSF) controls miR-29c expression.

## Methods

All animal work must have been conducted according to relevant national and international guidelines.

### *In vitro* ischemia

PC12 cells were purchased from American Type Culture Collection (ATCC; Manassas, VA, USA; Catalog # CRL-1721). PC12 cells were grown in Dulbecco's modified Eagle's medium supplemented with 4.5 mg/ml glucose, 5% bovine calf serum and 15% equine serum. On the day of the experiment, cells were washed twice with OGD buffer (glucose-free isotonic salt solution; 20 mM NaHCO_3_, 120 mM NaCl, 5.36 mM KCl, 0.33 mM Na_2_HPO_4_, 0.44 mM KH_2_PO_4_, 1.27 mM CaCl_2_ and 0.81 mM MgSO_4_; pH 7.4) [12], and incubated in fresh OGD buffer for 6 h in an OGD chamber (94% nitrogen, 5% carbon dioxide and <1% oxygen) at 37°C. The cells were then transferred to normoglycemic medium and incubated under normoxic conditions for 24 h at 37°C. As a control, PC12 cells were incubated under normoxic conditions in OGD buffer supplemented with 4.5 mg/ml glucose for 6 h and changed to normal medium and incubated for 24 h at 37°C.

### Focal Ischemia

Transient MCAO was induced in adult male spontaneously hypertensive rats (SHR; 280–300 g; Charles River USA) under isoflurane anesthesia by the intraluminal suture method as described earlier [13], [14]. All the surgical procedures were approved by the Research Animal Resources and Care Committee of the University of Wisconsin-Madison and the animals were cared for in accordance with the *Guide for the Care and Use of Laboratory Animals, U.S. Department of Health and Human Services Publication Number. 86–23* (*revised*). All surgery was performed under isoflurane anesthesia, and all efforts were made to minimize suffering. The left femoral artery was cannulated for continuous monitoring of arterial blood pressure and to obtain the measurements of pH, P_a_o_2_, P_a_co_2_, hemoglobin and blood glucose (i-STAT; Sensor Devices USA). The rectal temperature was controlled at 37.0±0.5°C during surgery with a feedback-regulated heating pad. After a midline skin incision, the left external carotid artery (ECA) was exposed, and its branches were coagulated. A surgical monofilament 3–0 nylon suture blunted at the end was introduced into the ECA lumen and gently advanced to the internal carotid artery until the regional cerebral blood flow (rCBF) was reduced to 13±3% of the baseline (recorded by laser Doppler flowmeter; Vasamedics LLC USA) as described earlier [15], [16]. The MCA was occluded transiently for 1 h. The restoration of the blood flow was confirmed by laser Doppler following the withdrawal of the suture. After suturing the wound, 0.5% bupivacaine (0.25 ml) was injected along the incision to provide short duration local anesthesia. The animals were allowed to recover from anesthesia and returned to the cage with *ad libitum* access to food and water. During the surgery, rats were under spontaneous respiration.

### Vectors and transfection

DNMT3a 3′UTR sequence was amplified from rat brain genomic DNA and cloned downstream to luciferase in pMIR-REPORT system (Ambion) at *Xho*I site by using Infusion Cloning Kit (Clonetech USA) as per manufacturer's instructions. A DNMT3a 3′UTR mutant plasmid was generated by creating a point mutation in the miR-29c binding site using the Site-Directed Mutagenesis Kit (Invitrogen USA) as per manufacturer's instructions. The miR-29c promoter (2 Kb) and 2 mutants (1.9 Kb and 1.1 Kb; lacking REST binding sites) were amplified from rat brain genomic DNA and cloned in pGL-3 basic vector (Promega USA). Sequences of the wild-type and mutant vectors were verified with restriction digestion and automated DNA sequencing. The REST vectors (pMT-REST and pMT-DN-REST) were same as those used before [17]. The pRenilla luciferase vector was from Promega USA.

### PremiR, antagomiR and *si*RNA treatment *in vitro*

PremiR-29c, antogomiR-29c, control premiR, control antagomiR, DNMT3a *si*RNA, REST *si*RNA and control *si*RNA (all from Ambion USA) were transfected into PC12 cells (1×10^6^ cells/well) at a concentration 1.50 µM using siPORT NeoFX transfection reagent (Invitrogen USA) according to the manufacturer's instructions. The DNMT3a *si*RNA and REST *si*RNA used were from Ambion USA and validated by the Silencer Select Algorithm (Ambion) and BLAST to avoid the possible targeting of other homologous genes. To account for the non-sequence-specific effects, an Ambion control non-targeting *si*RNA with comparable GC content to that of the functional *si*RNA but lacking identity with known gene targets and had at least 4 mismatches with all known human, mouse and rat genes was used. Each transfection was conducted in triplicate and each experiment was repeated 4 times. Twelve hours following transfection, cells were subjected to OGD and reoxygenation (6 h and 24 h) or incubated under normoxic conditions with glucose (control). The cell viability was analyzed by Trypan Blue exclusion assay.

### Luciferase reporter assays

PC12 cells (4×10^4^ cells/well) were transfected with wild-type or mutant pMIR-DNMT3a 3′UTR plasmid together with 20 nM or 100 nM premiR-29c or control premiR. In other experiments, cells were transfected with pGL3-miR-29c promoter plasmid or one of the mutant pGL3-miR-29c promoter plasmids (that lack REST binding sites) together with pMT-REST or pMT-dominant negative REST (DN-REST). Each plasmid was transfected at a concentration of 200 ng together with 100 ng Renilla luciferase plasmid (transfection control) using siPORT Neo FX. Two days after transfection, cells were lysed and subjected to a dual luciferase assay (Promega USA). Each transfection was conducted in triplicate and each experiment was repeated 4 times.

### Western blotting

Cells or brain tissue were homogenized in ice-cold 25 mM Tris-HCl buffer (pH 7.4) containing 2 mM EDTA and protease inhibitor cocktail (4-(2-aminoethyl) benzenesulfonyl fluoride, aprotinin, leupeptin, bestatin, pepstatin-A, and transepoxysuccinyl- L-leucylamido(4-guanidino)butane; Sigma Chemical Co USA). Proteins were solubilized by adding Lamelli electrophoresis sample buffer (5% sodium dodecyl sulfate, 20% glycerol, 10% 2- mercaptoethanol, 125 mmol/L Tris-HCl, pH 6.8, and 0.004% bromophenol blue; Sigma Chemical Co. USA) and denatured by heating at 94°C for 3 min. Samples (20-25 µg protein equivalent) were electrophoresed on 4–20% polyacrylamide gradient gels (Bio-Rad USA Criterion precast gels), transferred to PVDF membranes and probed with polyclonal DNMT3a (1∶1,000, Cell Signaling USA), polyclonal REST (1∶1,000; Santa Cruz USA) and monoclonal β-actin (1∶2,000, Cell Signaling USA) antibodies followed by HRP-conjugated anti-rabbit or anti-mouse IgG (1∶2,500). The protein bands recognized by the antibodies were visualized by enhanced chemiluminescence according to the manufacturer's instructions (Pierce USA).

### Real-time PCR

Real-time PCR was conducted as described earlier using the SYBR-green method [3]. The reverse transcription was performed using the TaqMan® MiRNA Reverse Transcription Kit (Applied Biosystems USA) and the PCR reactions were performed using the TaqMan® MiRNA Assay Kit (Applied Biosystems USA) as per manufacturer's instructions. The threshold cycle (*C*_t_) method (http://pebiodocs.com/pebiodocs/04303859.pdf) was used to determine the relative quantities of each miRNA. The sequences (5′–3′) of the amplified miRNA transcripts are AUU GGC UAA AGU UUA CCA CGA U(rno-miR-29c) and UGA GGU AGU AGG UUG UAU AGU U (rno-Let-7a). Let-7a was used as a control as it is one of the highly expressed miRNAs in rat brain which was observed to be unaltered after focal ischemia (Dharap et al., 2009). GAPDH and 18S rRNA were used as additional internal controls and their primer sequences are same as in a previous study (Dharap et al., 2009).

### *In vivo* administration of premiRs and *si*RNAs

PremiR-29c or control premiR (both from Ambion USA) were infused into rat brain lateral ventricles as described previously [3]. In brief, the premiRs were dissolved in artificial CSF (aCSF; 119 mM NaCl, 3.1 mM KCl, 1.2 mM C_a_Cl_2_, 1 mM MgSO_4_, 0.50 mM KH_2_PO_4_, 25 mM NaHCO3, 5 mM D-glucose, 2.2 mM urea, pH 7.4) to obtain a concentration of 5 µM and filled into osmotic minipums (Alzet model 1030D that pump at a rate of 1 µL/h; Alza USA). Each pump was connected to an Alzet brain infusion stainless steel cannula by peristaltic tubing and primed for 4 h at 37°C. The cannula was stereotaxically implanted into the lateral ventricle [bregma; 0.8 mm posterior, −4.8 mm dorsoventral, −1.5 mm lateral; based on the rat brain atlas of Paxinos and Watson (1998)] and secured to the skull with dental cement. The pump was placed in the skin fold on the neck of the rat. The cannula and pump implantation was conducted under isoflurane anesthesia. Rats were subjected to 1 h transient MCAO and cohorts were killed either at 1 day of reperfusion to estimate DNMT3a protein levels (n = 4/group) or 3 days of reperfusion to estimate infarct volume (n = 8/group).

Intracerebral injection of *si*RNAs was conducted as described earlier [18]. In brief, the in vivo grade DNMT3a *si*RNA or control *si*RNAs was suspended in *si*RNA universal buffer to yield a concentration of 740 µm and 8 µL of this was combined with 2 µL of oligofectamine and incubated at room temperature for 15 min. The *si*RNAs were injected slowly using a Hamilton syringe into the cerebral cortex [bregma; −0.2 mm posterior, 3 mm dorsoventral, 4.5 mm lateral; based on the rat brain atlas of Paxinos and Watson (1998)]. Rats were subjected to 1 h transient MCAO and cohorts were killed either at 1 day of reperfusion to estimate DNMT3a protein levels (n = 4/group) or 3 days of reperfusion to estimate infarct volume (n = 8/group).

### Infarct volume estimation

Infarct volume was measured as described earlier [3], [15], [16], [18]. Each rat was perfused transcardially with buffered paraformaldehyde, the brain was post-fixed, cryoprotected and sectioned (coronal; 40 µm thick at an interval of 320 µm). The serial sections were stained with Cresyl violet and scanned using the NIH Image program. The volume of the ischemic lesion was computed by the numeric integration of data from 6 serial sections from each rat in respect to the sectional interval. To account for edema and differential shrinkage resulting from tissue processing, the injury volumes were corrected by using the Swanson formula: corrected injury volume  =  contralateral hemisphere volume – (ipsilateral hemisphere volume - measured injury volume) [19].

## Results

### PremiR-29c treatment prevented ischemic cell death

**Figure 1:**
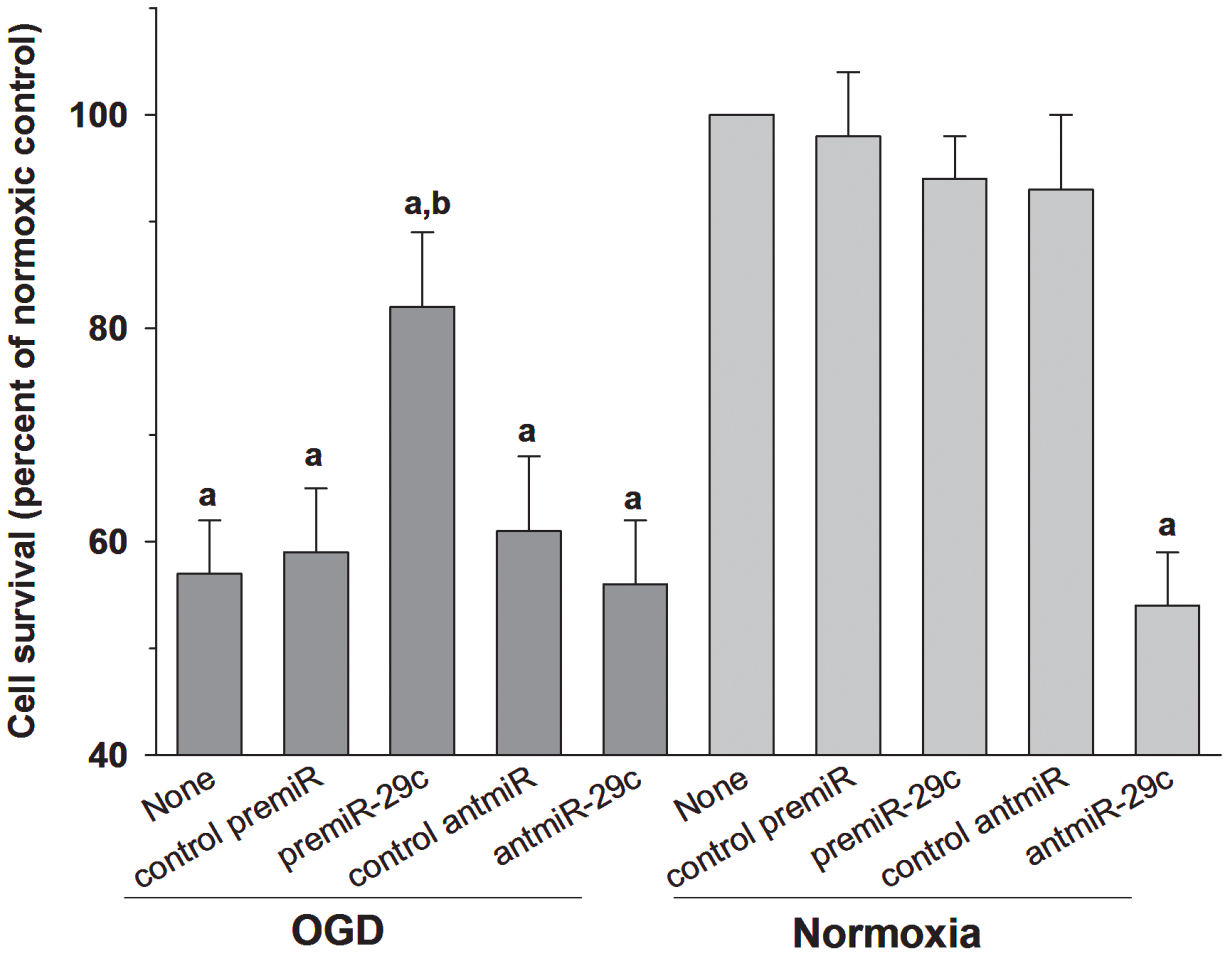
Treatment of PC12 cells with premiR-29 significantly curtailed OGD-induced cell death. AntagomiR-29c treatment had no effect on post-OGD cell death. On contrary, under normoxic conditions, antagomiR-29c treatment induced cell death, but premiR-29c treatment had no effect. Values are mean ± SD (n = 4/group) of triplicate experiments. Statistics: ^a^p<0.05 compared to normoxia/none group and ^b^p<0.05 compared to the OGD/none group.

When PC12 cells were subjected to OGD, the miR-29c levels decreased significantly (by -2.4 fold; p<0.05) compared to normoxic control by 24 h (Table 1). Treatment with premiR-29c (for 12 h) increased the post-OGD miR-29c levels by 3.6 fold over control premiR treated group (Table 1). OGD induced a 43±7% cell death in PC12 cells by 24 h (Fig. 1). OGD-induced cell death was only 18±5% in the premiR-29c treated cells, whereas control premiR treated group showed a 47±9% cell death (Fig. 1). AntagomiR-29c treatment had no significant effect on post-OGD cell death compared to untreated or control antagomiR treated groups (Fig. 1). On the contrary, under normoxic conditions incubation with antagomiR-29c for 12 h induced a 46±6% cell death compared to control antagomiR treated group (Fig. 1). But, premiR-29c had no significant effect on cell survival under normoxic conditions (Fig. 1). Thus restoring miR-29c levels protects against OGD, while providing excess miR-29c had no effect under normal conditions. Furthermore, knocking-down miR-29c promotes cell death under normal conditions. These observations show that miR-29c is a prosurvival miRNA under physiological conditions and its down-regulation after ischemia is a promoter of cell death.

**Table 1:** PremiR-29c and REST siRNA treatment restored post-ischemic levels of miR-29c.

OGD	−2.4±0.2^a^
OGD + control premiR	−2.1±0.3^a^
OGD + premiR-29c	3.6±0.6^a,b^
OGD + control *si*RNA	−2.0±0.3^a^
OGD + REST *si*RNA	2.9±0.5^a,b^

The PC12 cells were incubated with the premiRs or *si*RNAs for 12 h and then subjected to OGD for 6 h followed by incubation under normoxic/normoglycemic conditions for 24 h. The miR-29c levels were estimated with real-time PCR. The values are given as fold change over the normoxic/normoglycemic control group. Let-7 and 18 s rRNA used as housekeeping controls were unaltered by any of the treatments. Values are mean ± SD of n = 4/group. Each PCR reaction was conducted in triplicate. Statistics: ^a^p<0.05 compared to normoxic/normoglycemic control, and ^b^p<0.05 compared to respective OGD+control group (one-way ANOVA followed by Neuman-Keul's multiple comparisons post-test).

### DNMT3a is a miR-29c target

**Figure 2:**
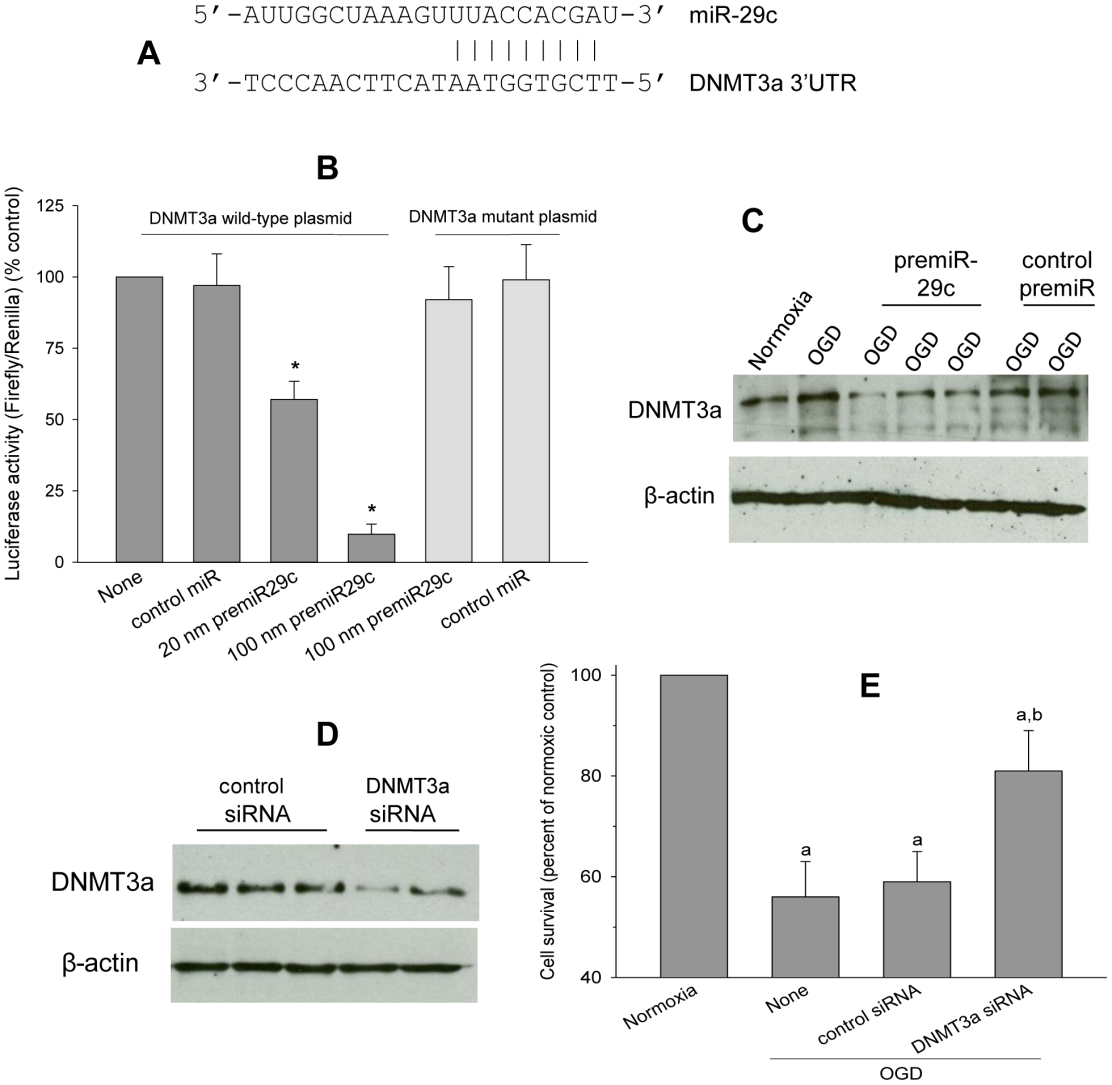
In PC12 cells, OGD-induced cell death was mediated by miR-29c down-stream target DNMT3a. The miR-29 seed sequence in the 3′UTR of DNMT3a mRNA (A). Treatment with premiR-29c dose-dependently prevented the expression of DNMT3a 3′UTR vector compared to control miR treatment (B). PremiR-29c had no effect on the expression of the DNMT3a 3′UTR mutant vector containing a point mutation in the miR-29c binding site compared to control miR treated group (B). OGD increased DNMT3a protein levels in PC12 cells compared to normoxic control (C). Treatment with premiR-29c decreased post-OGD DNMT3a protein levels compared to control miR treatment (C). Protein levels of β-actin used as a loading control were unaltered by OGD or premiR treatment (C). Post-OGD DNMT3a protein levels were knocked-down in the PC12 cells treated with DNMT3a *si*RNA compared with control *si*RNA treated group (D). Treatment with DNMT3a *si*RNA also decreased OGD-induced cell death significantly compared to control *si*RNA treatment (E). Values in the histograms are mean ± SD (n = 4/group) of triplicate determinations.

Using TargetScan and Microcosm from MiRBase, we analyzed the targets of rat miR-29c. Targetscan predicted 733 targets while Microcosm predicted 1,176 targets and 135 of those are conserved targets predicted by both algorithms. Of those, we excluded 87 as weak targets or false positives based on score and energy. DNMT3a is one of the predicted miR-29c targets identified by both algorithms with a very high score and energy. The miR-29c seed sequence of DNMT3a was shown in Fig. 2A. We crosschecked the target-miRNA relationship using DNMT3a as an input mRNA in Microcosm which showed that rat DNMT3a is targeted by only 2 miRNAs (rno-miR-29c and rno-miR-300-3p) of which rno-miR-300-3p was unaltered after MCAO [3].

To conclusively show the miR-target relationship, we co-transfected DNMT3a 3′UTR vector and a mutant DNMT3a 3′UTR vector (premiR-29c seed sequence was mutated; control) with premiR-29c in PC12 cells. The premiR-29c prevented DNMT3a 3′UTR expression (20 nmol and 100 nmol of premiR-29c prevented the luciferase activity by 37% and 84%, respectively; Fig. 2B). Disruption of the miR-29c binding site completely abrogated the inhibition of luciferase activity by premiR-29c (Fig. 2B). Renilla luciferase was used to normalize the values.

#### PremiR-29c prevented post-OGD DNMT3a protein induction

Compared to normoxic control (lane 1 of Fig. 2C), DNMT3a protein levels increased significantly after OGD (lane 2 of Fig. 2C). When cells were treated with premiR-29c (1.5 µM) for 12 h before subjecting them to OGD, DNMT3a protein induction was significantly curtailed (lanes 3 to 5 of Fig. 2C) compared to control miR treatment (lanes 6 and 7 of Fig. 2C).

#### DNMT3a *si*RNA decreased OGD-induced cell death

When PC12 cells were incubated with DNMT3a *si*RNA or control *si*RNA for 12 h and then subjected to OGD, the DNMT3a *si*RNA treated group showed significantly lower post-OGD DNMT3a protein levels (lanes 4 to 6 of Fig. 2D) compared to control *si*RNA treated group (lanes 1 to 3 of Fig. 2D). DNMT3a *si*RNA treatment also significantly prevented the post-OGD cell death (by 54±6%; p<0.05) compared to control *si*RNA treated group (Fig. 2E).

### Treatment with premiR-29c or DNMT3a *si*RNA decreased infarction after MCAO

**Figure 3:**
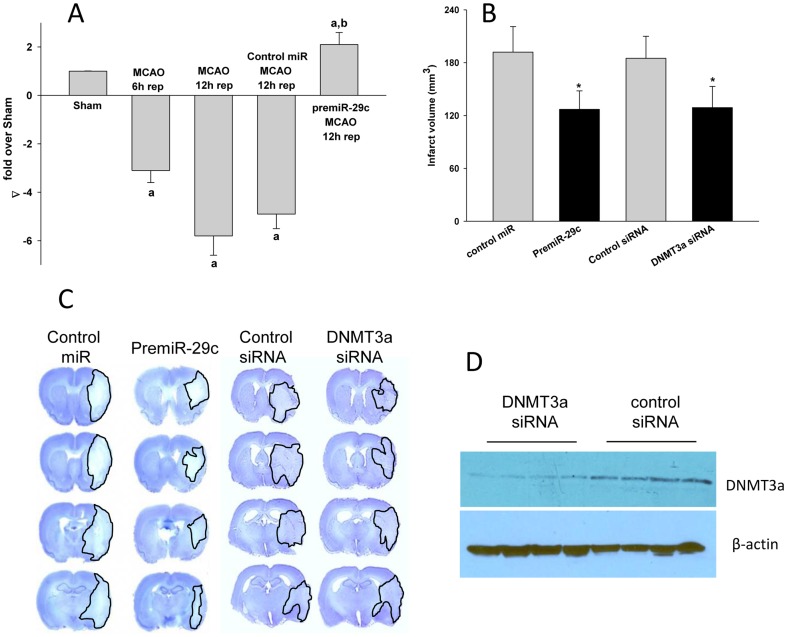
miR-29c and DNMT3a contributes to the development of focal ischemia-induced infarction in rat brain. Transient MCAO significantly decreased miR-29c levels in the ipsilateral cortex of rats at 12 h reperfusion (A) and treatment with premiR-29c led to significantly increased post-ischemic miR-29c levels compared to control miR treated group (A). The miR-29c levels were measured by real-time PCR using 18 s rRNA and Let-7a as house-keeping controls. Compared to control miR treated group, premiR-29c treated group showed a significantly decreased infarct volume measured at 3 days of reperfusion following a 1 h transient MCAO in adult rats (B). Treatment with DNMT3a *si*RNA also significantly decreased the post-MCAO infarct volume compared to control *si*RNA treated group in adult rats (B). Treatment with DNMT3a *si*RNA significantly decreased the post-MCAO DNMT3a protein levels compared to control *si*RNA treated group (C). Cresyl violet stained serial brain sections from representative rats of control miR, premiR-29c, control *si*RNA and DNMT3a *si*RNA treated groups are shown in Panel D. Values in the histograms are n = 4/group for panel A and n = 8/group for panel B. ^a^p<0.05 compared with the sham group, ^b^p<0.05 compared with the control premiR treated group and *p<0.05 compared with the respective control group (one-way ANOVA followed by Neuman-Keul's multiple comparisons post-test).

As the *in vitro* studies showed that miR-29c down-regulation is a proponent of ischemic cell death, we evaluated if providing premiR-29c decreases focal ischemia-induced brain damage *in vivo*. Using real-time PCR, we presently observed that miR-29c was down-regulated by 5.8±1.1 fold in the ipsilateral cortex of rats subjected to transient MCAO and 12 h reperfusion compared to sham control (Fig. 3A). These studies confirm our previous microarray studies that showed miR-29c down-regulation after focal ischemia (Dharap et al., 2009). Whereas, rats injected with premiR-29c, but not control miR, subjected to transient MCAO showed a complete recovery of miR-29c levels by 12 h of reperfusion (Fig. 3A). Furthermore, the premiR-29c injected rats showed a significantly decreased infarct volume (by 34%±6%; p<0.05; n = 8/group) compared to control miR injected rats (Fig. 3B). The rCBF, arterial blood pressure and the other physiological parameters measured during the MCAO and reperfusion were not significantly different between the premiR-29c and control miR injected groups (data not shown).

As the *in vitro* studies showed that DNMT3a is a mediator of the miR-29c down-regulation induced ischemic cell death, we tested the role of DNMT3a in post-ischemic brain damage following transient MCAO. When rats were treated with DNMT3a *si*RNA, the post-ischemic ipsilateral cortical DNMT3a levels were significantly lower (by 81%±13%; p<0.05; n = 4/group) than the control siRNA treated group at 24 h reperfusion (Fig. 3C). The DNMT3a *si*RNA treated rats also showed significantly smaller infarct volume compared to control *si*RNA treated rats (by 30%±5%; p<0.05; n = 8/group) (Fig. 3B).

#### REST curtailed miR-29c promoter expression

**Figure 4:**
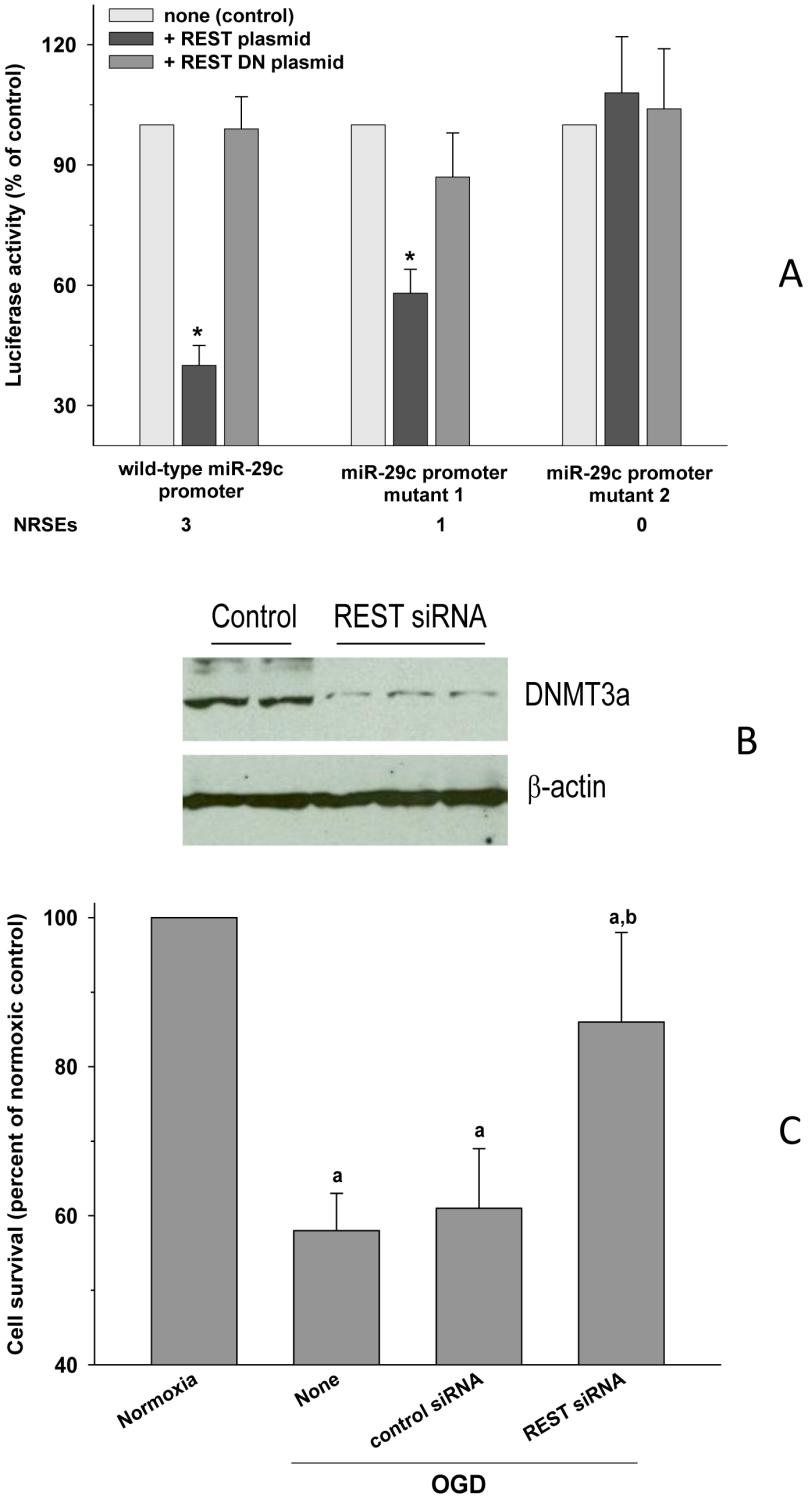
REST mediates miR-29c expression after ischemia. The miR-29c promoter has 3 upstream binding sites within 1 Kb from TSS for the transcription factor REST. Transfection with a REST plasmid inhibited miR-29c promoter vector expression which was ablated by deletion of REST binding sites (A). Treatment with REST *si*RNA curtailed the post-OGD DNMT3a protein expression compared to a control *si*RNA treated group (B). REST *si*RNA treatment also minimized the OGD-induced cell death compared to control *si*RNA treatment (C). Values in the histograms are n = 4/group. In panel A, *p<0.05 compared with none (control) group. In panel C, ^a^p<0.05 compared with the normoxia group and ^b^p<0.05 compared with the none/OGD group. Statistical analysis was conducted with one-way ANOVA followed by Neuman-Keul's multiple comparisons post-test.

To understand the factors that control miR-29c after ischemia, we analyzed the transcription factor binding sites in the miR-29c gene promoter using the Genomatix search algorithm (Genomatix GmbH). We identified 3 binding sites for the transcription factor REST within 1 Kb from transcription start site (TSS) located at −2 to −32, −94 to −124 and −838 to −868 upstream from TSS on the same strand of the DNA from which miR-29c is transcribed. Cotransfection with REST plasmid inhibited miR-29c promoter (2 Kb rat miR-29c promoter cloned in Promega promoterless pGL-3 basic vector) expression in PC12 cells by 62±5% compared to dominant negative (DN) REST plasmid transfected group (Fig. 4A). The REST mutant plasmids which are 1.9 Kb and 1.1 Kb; lacking either 2 REST binding sites or all 3 REST binding sites showed ablation of the REST-mediated suppression of miR-29c promoter by 45 and 100%, respectively compared to DN REST transfected control (Fig. 4A). This indicates that the REST binding sites in the miR-29c promoter can control miR-29c expression.

#### REST siRNA decreased ischemic cell death

When PC12 cells were incubated with REST *si*RNA (1.5 µM for 12 h) and then subjected to OGD, miR-29c levels increased by 2.9 fold compared to control *si*RNA treated group (Table 1). The REST *si*RNA treated group also showed a significantly lower post-OGD DNMT3a protein levels (lanes 3 to 6 of Fig. 4B) compared to control *si*RNA treated group (lanes 1 and 2 of Fig. 4B). REST *si*RNA also significantly reduced the cell death (by 64±9%; p<0.05) in PC12 cells subjected to OGD compared to control *si*RNA (Fig. 4C).

## Discussion

Despite decades of experimentation, no drugs that can prevent post-ischemic brain damage in humans have emerged. One of the reasons for this might be the focus on therapeutic targeting of various classes of proteins that modulate post-stroke pathophysiology. In mammals, only <2% of the genome transcribes protein-coding RNAs (mRNAs) while the >70% of the genome transcribes non-coding RNAs [20]. As various classes of non-coding RNAs that include miRNAs are emerging as the master controllers of transcription and translation, their functional changes might be a factor in deciding the pathological outcome after stroke [1]. Hence, understanding the mechanism of action of miRNAs and their dynamic interactions with down-stream targets and upstream transcription factors will open new avenues for therapeutically targeting the secondary brain damage.

Recent studies showed that stroke alters miRNA expression profiles in rodents as well as humans [3], [21], [22], [23]. Importantly, the miRNAome responds rapidly and many of the changes sustain through the progression of the brain damage (the first 3 days) following stroke (Dharap et al., 2009). Our lab showed that knocking-down miR-145, a highly stroke-induced miRNA increases the expression of its down-stream target superoxide dismutase-2 with concomitant neuroprotection in rats subjected to transient MCAO [3]. A subsequent study showed that miR-145 also targets the anti-inflammatory cytokine IFN-β that is known to decrease infarction by attenuating the neutrophil and macrophage infiltration into the ischemic brain [24]. Hence, miR-145 induction seems to curtail the ability of the brain to mitigate oxidative stress and inflammation thus contributing to the post-ischemic brain death. Knocking-down other stroke-induced miRNAs miR-497 and miR-15a was also reported to be beneficial by increasing the expression of their common target Bcl2 which is an important anti-apoptotic protein [10], [11]. Furthermore, upregulation of miR-21 was also shown to prevent the translation of Fas ligand leading to decreased post-ischemic apoptosis [2].

The miR-29c is one of the highly expressed miRNAs in rat brain. Furthermore, transient focal ischemia led to a sustained silencing of miR-29c during the acute phase of reperfusion (2 h to 3 days) during which the cell death is on-going but can be prevented with timely intervention. We hypothesize that a highly expressed miRNA like miR-29c might play an essential role in maintaining the cellular homeostasis in adult brain and its dysfunction contributes to ischemic brain damage. Our data support this hypothesis by showing that restoring miR-29c levels by treatment with premiR-29c is neuroprotective after ischemia. We also observed that PC12 cells die if miR-29c is knocked-down with an antagomiR-29. Thus, miR-29c might be essential for cell survival under homeostatic conditions and its down-regulation after ischemia is derogatory. Our studies also showed that replenishing miR-29c with premiR-29c treatment significantly decreases the infarct volume after transient MCAO confirming the role of miR-29c as an endogenous pro-survival miRNA under *in vivo* conditions as well.

Being non-coding, the major function of a miRNA is to silence its target mRNAs from translating their protein products. Hence the pathological effects of a miRNA down-regulated after an insult are mediated by de-repression of one or more of its down-stream targets.

In silico analysis showed high score and a low mean free energy for DNMT3a and miR-29c interaction indicating that DNMT3a is a robust target of miR-29c. We confirmed the miR-target relationship experimentally using DNMT3a 3′UTR luciferase vector assays by challenging with premiR-29c. While DNMT1 is a maintenance methyltransferase, DNMT3a and DNMT3b are *de novo* methyltransferases [25], [26]. As aberrant methylation can shut-down cripple the normal transcriptional activity, maintaining a proper rate of DNA methylation is a prerequisite for cellular homeostasis. Based on our results we hypothesize that controlling DNMT3a might be a conserved function of a highly expressed miRNA like miR-29c that leads to brain damage if disturbed. Following ischemia, down-regulation of miR-29c might derepress DNMT3a translation resulting in increased amounts of DNMT3a protein as observed in the present study. We also observed that knocking-down DNMT3a protein with DNMT3a *si*RNA decreased infarction in vivo and cell death in vitro indicating that DNMT3a induction is a potential mechanism of miR-29c down-regulation mediated ischemic damage.

Understanding the upstream mechanisms that control the expression of a miRNA helps manipulating miRNAs (and thus their down-stream targets) under pathological conditions with a goal to improve the functional outcome. Bioinformatics showed 3 binding sites for the transcription factor REST in the miR-29c putative promoter within 1 Kb upstream to miR-29c coding region in the same strand of DNA from which miR-29c is transcribed. This suggests REST as a potential transcriptional controller of miR-29c. We confirmed this by experimentally showing that a REST expressing plasmid curtailed the miR-29c promoter plasmid expression and this inhibitory effect was removed when the REST binding sites in the miR-29c promoter were mutated. As REST is a suppressor of gene expression, its low levels might allow high expression of miR-29c in normal adult brain. A recent study showed that global ischemia activates REST and its knockdown protects hippocampal neurons from ischemic cell death [27]. We observed that treatment with REST *si*RNA significantly increased the levels of miR-29c, curtailed DNMT3a protein induction and decreased the cell death following ischemia. This indicates that REST induction might be responsible for miR-29c suppression (and thus derepression of DNMT3a) leading to ischemic brain damage. The contribution of other targets of miR-29c to ischemic brain damage can't be ruled out from the present studies, but our data firmly establishes miR-29c as a prosurvival miRNA that is under REST control and disruption of the REST, miR-29c and DNMT3a homeostasis is one of the mediators of post-stroke brain death.
